# Insoluble fibers affect digesta transit behavior in the upper gastrointestinal tract of growing pigs, regardless of particle size

**DOI:** 10.1093/jas/skad299

**Published:** 2023-09-04

**Authors:** Sebastian Dorado-Montenegro, Kim Lammers-Jannink, Walter Gerrits, Sonja de Vries

**Affiliations:** Animal Nutrition Group, Wageningen University & Research, Wageningen, Gelderland, 6700 AH, The Netherlands; Escuela de Zootecnia, Universidad de Costa Rica, San José 2060, Costa Rica; Animal Nutrition Group, Wageningen University & Research, Wageningen, Gelderland, 6700 AH, The Netherlands; Animal Nutrition Group, Wageningen University & Research, Wageningen, Gelderland, 6700 AH, The Netherlands; Animal Nutrition Group, Wageningen University & Research, Wageningen, Gelderland, 6700 AH, The Netherlands

**Keywords:** cannulated pigs, markers, mean retention time, mordants, oat husks, soybean hulls

## Abstract

Physicochemical characteristics of dietary fibers may modulate digesta transit behavior. The present study was conducted to clarify the effect of level and particle size (PS) of insoluble fibers on digesta mean retention time (MRT) in the proximal gastrointestinal tract (mouth-ileocecal junction). Six ileal-cannulated pigs (26.8 ± 2.08 kg) were assigned to 3 dietary treatments in a 3 × 3 replicated Latin-square design. Finely ground (1 mm screen) or coarse (intact) oat husks (OH) and soybean hulls (SBH) were added (50:50, w/w) to a maize–whey protein–wheat-based diet at 50 (low) or 250 g/kg (high) inclusion levels to obtain a low-fine fiber (LF), high-fine fiber (HF), and high-coarse fiber (HC) diet. Markers to follow liquids (Co-EDTA), fine solids (Y_3_O_2_), or fibrous particles (Yb-mordanted OH and Cr-mordanted SBH) were given as a single pulse dose and marker concentrations were subsequently measured hourly in digesta for 13 h after administration. Mean retention time values were obtained from the concentration of markers in digesta observed over time by fitting a generalized Michaelis–Menten equation and calculating the time of peak. Fiber addition and fiber particle size neither affected the MRT of liquid nor solid digesta phases (*P* = 0.903). Segregation between solid and liquid digesta phases was observed for all diets (*P* < 0.0001), although the extent of segregation was greater for LF compared with HF and HC (*P* = 0.0220). The MRT of SBH particles, but not of OH-particles was longer for coarse vs fine PS (96 min, *P* < 0.05). In conclusion, digesta MRT was influenced by the dietary concentration but not by PS of insoluble fibers. The addition of insoluble fibers reduces digesta phase segregation from mouth to distal ileum in growing pigs.

## Teaser

This paper gives insights into the effect of addition of insoluble fibers varying in particle size on digesta transit along the proximal gastrointestinal tract of growing pigs. These insights are relevant for diet formulation as they can explain variations in nutrient digestion and absorption kinetics.

## Introduction

The pressure to increase the use of fibrous ingredients in human and animal food has led to greater attention to their effect on digestive processes. The type and quantity of non-starch polysaccharides (NSP) present in cell walls of natural fibrous feedstuffs vary widely (Bach Knudsen, 1997) and so does their possible impact on digestion. Insoluble fibers are known to play a role in regulating digesta transit behavior and nutrient digestibility in pigs (Gutierrez et al., 2013; Agyekum and Nyachoti, 2017).

Variation in the concentration of insoluble fibers can alter the transit of digesta phases in different ways along the gastrointestinal segments (Guerin et al., 2001; Van Leeuwen et al., 2006; Wilfart et al., 2007), and nutrient digestibility (Zhang et al., 2013). Altering the retention time of solid or liquid digesta can result in changes in digesta phase segregation, potentially impacting nutrient digestion kinetics (Zijlstra et al., 2012; Martens et al., 2018). In this regard, Gerrits et al. (2021) stressed the importance of modelling digestion kinetics as a next step in development of new feed ingredient evaluation system. For this, it is essential to gain a deeper understanding of how individual ingredients influence factors such as digestion potential, intestinal conditions, and digestion behavior.

Particle size of dietary components can also influence digesta transit behavior. In this regard, Bornhorst et al. (2013a) indicated that large digesta particles with slow breakdown rates tend to stay longer in the stomach to be reduced in size by gastric sieving before leaving the stomach. In their study, rice bran layers of brown rice diets were retained for longer time in the stomach compared to the particles of white rice diets. Vincent et al. (1995) observed similar results when coarse wheat bran (>200 mm) presented a longer gastric half emptying time in comparison with fine wheat bran (< 71 mm) and a rice-based control diet, in healthy young-adult humans. This study reported no differences in half-emptying time in the small intestine among diets.

The use of indigestible markers is common practice in studies of digesta transit behavior and digestibility. In such studies, it is assumed that the marker used transits parallel with the material of interest (de Vries and Gerrits, 2018). However, when digesta segregation occurs, particularly within the solid phase, it is possible that the marker no longer accurately represents the transit behavior of some digesta components. The above since larger particles remain in the stomach for a longer duration (Martens et al., 2019) and commonly used fine particle markers mainly represent the small solid particle fraction. Thus, the use of a combination of soluble, fine solid particle, and fibrous markers is recommended to get a better overview of the behavior of all digesta fractions.

Currently, there is a lack of clarity regarding how the level and particle size of a blend of insoluble fibers relates to the transit behavior of individual digesta phases in pigs. We hypothesized that an increase in dietary insoluble fibers will increase the retention time of solid digesta, increasing digesta phase segregation, especially for coarse fibers. Therefore, this study aimed to elucidate the effect of level and particle size of insoluble fibers on digesta transit behavior in the proximal gastrointestinal tract (mouth-terminal ileum) of growing pigs.

## Material and Methods

### Animals and housing

The experiment was approved by the Dutch Central Authority for Scientific Procedures on Animals (CCD, 2017.W-0025.005) and performed at the Research Facilities of Wageningen University & Research, (Wageningen, The Netherlands). This study was part of a larger animal experiment focusing on the interactive effect of insoluble fibers and protein infusions on colonic fermentation in pigs (Lammers-Jannink et al., unpublished).

A total of 10 barrows (initial BW 22.9 ± 1.9 kg (SD); Topigs Norsvin TN Tempo) were randomly assigned to one of two pens (5 pigs/pen). After a 13-day socialization period, 8 pigs were selected, based on docility and body conformation (BW of 26.8 ± 3.1 kg). They were surgically fitted with a T-cannula of 2.24 cm inner diameter placed 10 cm before the ileum–cecal junction, based on the protocol of Hodgkinson et al. (2020), and a subcutaneous catheter was placed to infuse protein products directly into the colon, as part of another experiment. The pigs were individually housed in metabolism cages (1.15 × 1.35 m), equipped with slatted floor, a single restricted-access feeder and an adjustable nipple drinker. Transparent walls between cages were installed to warrant social interactions among animals and toys were exchanged every 3 d as enrichment.

### Experimental design and diets

After 10 d of recovery, 6 pigs were assigned to 3 dietary treatments offered in 3 consecutive periods, using a 3 × 3 double Latin square design. The 2 extra animals served as reserve animals. Along the experiment 3 animals presented cannula displacement, one lost the cannula, and one presented feed refusals (>90%) during the first digesta collections, and these data were excluded from the experiment. Also, one pig presented a severe obstruction of the cannula so the data collected were not considered. Therefore, the experimental period was extended to collect enough observations. In total, 16 observations were obtained during 5 periods in an incomplete 8 × 5 Youden square. Each period consisted of 5 d of diet acclimatization, followed by 3 d of digesta collection of which the last collection day was used to study digesta transit behavior. The last period consisted of only 3 d of acclimatization to the diets.

Diets were offered with water in a 2:1 feed:water ratio, twice per day (07:30 and 16:30 hours) at a feeding level of 2.6 × net energy requirement for maintenance (290 kJ/kg BW^0.75^; Everts, 2015). At the beginning of each period, the feed allowance was adjusted to the body weight of the pigs. Pigs had ad libitum access to water during the whole experiment. At the end of measurements, all pigs were dissected to evaluate the condition of the cannula and surrounding intestinal tissue.

The 3 formulated diets differed in the inclusion level (low and high) and particle size (coarse and fine) of 2 fibrous ingredients—soybean hulls (SBH) and oat husks (OH) (Table 1). To reach a fine particle size, both ingredients were ground with a hammermill (LHM20/16, 1.5 kW; Condux International, Mankato, USA) equipped with a 1-mm screen, at 1,500 rpm. Diets contained 50 g/kg fine fiber hulls (25 g/kg SBH + 25 g/kg OH) for the low-fine diet (LF) or 250 g/kg of fine or coarse hulls (125 g/kg SBH + 125 g/kg OH) for the high-fine diet (HF) and high-coarse diet (HC), substituting maize starch, wheat gluten meal, soy protein concentrate, and calcium carbonate (Table 1).

**Table 1.** Ingredient and nutrient composition of experimental diets fed to growing pigs^1^^,^^2^

Item	Control diet	Experimental diets
	LF	HF, HC
*Ingredient composition (% in diet)*		
Wheat	23.5	23.5
Corn	21.1	21.1
Corn starch	19.47	1.63
Whey protein isolate	11.5	11.5
Sugar	10	10
Sunflower oil	3	3
Soybean hulls	2.5	12.5
Oat husks	2.5	12.5
Calcium carbonate	1.54	1.38
Monocalcium phosphate	1.54	1.54
Soy protein concentrate	1.4	0
Wheat gluten meal	0.6	0
Premix (vitamin + mineral)^*^	0.5	0.5
Titanium oxide	0.4	0.4
Salt	0.2	0.2
Magnesium sulfate	0.15	0.15
Potassium carbonate	0.1	0.1
l-Lysine HCl	0	0
dl-Methionine	0	0
l-Threonine	0	0
l-Tryptophan	0	0
*Calculated nutritional composition (g/kg)* ^a^		
Dry matter (DM)	901	903
Crude protein (CP)	176	175
Crude fat	46	50
Crude ash	45	53
Starch	490	334
Dig. lysine	11.5	11.9
Dig. methionine	3.1	3.0
Dig. threonine	9.0	8.7
Dig. tryptophan	2.2	2.1
Non-starch polysaccharides (NSP)	108	243
NE (MJ/kg)^a^	11.5	9.9

^1^LF, finely ground low-fiber diet, contained 5% fine fibers (2.5% OH + 2.5% SBH).

^2^HF and HC, finely ground (1 mm screen) high-fiber diet and coarse (intact) high-fiber diet, contained 25 % (12.5% OH + 12.5% SBH) of fine and coarse fibers, respectively.

^a^Calculated based on the composition and nutritional value of feed materials (CVB, 2018).

^*^Composition of premix/kg diet: 10,000 IU vit. A, 2,000 IU vit. D3, 40 mg vit. E, 1.5 mg vit. K3, 1.0 mg vit. B1, 4.0 mg vit. B2, 1.5 mg vit B6, 20 μg vit. B12, 30 mg niacin, 15 mg d-pantothenic acid, 150 mg choline chloride, 0.4 mg folic acid, 0.05 mg biotin, 100 mg Fe (as FeSO_4_ H_2_O), 20 mg Cu (as CuSO_4_ 5H_2_O), 30 mg Mg (as MnO), 70 mg Zn (as ZnSO_4_ H_2_O), 0.7 mg I (as KI), 0.25 mg Se (as Na_2_SeO_3_). Carrier: milled corn.

### Ileal digesta collections

On the last day of each period, the pigs received the morning meal mixed with a pulse dose of 3.5 g of Y_2_O_3_ (solid-phase marker) and 3.5 g of Co-EDTA (liquid-phase marker). In addition, this meal contained a pulse dose of the mordanted fibers mixed with the HF or the HC diet to achieve similar intakes of all nutrients compared with the original treatment period. To this end, this meal contained 80% of the LF diet and 10% of each fine or coarse fiber source, i.e., 4% Cr-mordanted SBH + 4% C36-coated SBH + 2% regular SBH; 4% Yb-mordanted OH + 4% C32-coated OH + 2% regular OH; to reflect the nutritional composition of the HF and HC diets. The mordanting procedure was based on Udén et al. (1980) with some modifications. Fibers were not treated with sodium lauryl sulfate, and the washing procedure to eliminate soluble green matter involved 3 rounds, both before and after adding ascorbic acid, of centrifugation at 3,000 × *g* for 10 min and water extraction using a vacuum.

Half an hour before sampling began, pigs were fed and all digesta present in the cannulas was removed. Digesta samples were collected at 30, 90, 150, 210, 270, 330, 390, 450, 510, 570, 630, 690, and 750 min after the meal. When there was no sample in the bag at time of collection, sampling was continued for maximum 15 min; the actual collection time was recorded. After each collection, cannulas were closed, samples were weighted, and the bags were immediately frozen (−20 °C). When obstruction of the cannula occurred, digesta was taken out by gentle manipulation, and cannulas were flushed with demi water.

### Analytical methods

Prior to chemical analyses, digesta samples were freeze-dried first. Then, feed and digesta samples were ground by a centrifugal mill (Retsch ZM200, Haan, Germany) at 12,000 rpm using first a 1-mm and then a 0.5-mm screen. An extra grinding step was performed using a ball mill (Retsch MM 200, Haan, Germany) at a frequency of 30 Hz to address the presence of coarse OH particles that persisted after centrifugal grinding. Each sample underwent 3 cycles of 1-min grinding and 1-min interval to ensure the sample’s burning. Next, all samples were destructed using potassium bromate and phosphoric manganese sulfate solutions following the procedure presented by Williams et al. (1962). The concentrations of cobalt, yttrium, chromium, and ytterbium were analyzed using an Avio 500 inductively coupled plasma optical emission spectrometer (Perkin Elmer, Waltham, USA; van Bussel et al., 2010). Randomly, 10% of the samples were analyzed in duplicate to evaluate the precision of the method.

Particle size distribution of the fibrous ingredients was measured in duplicate by dry sieving (ASABE, 2008). In brief, one hundred grams of sample were sieved for 10 min trough six weighted sieves (2.5, 1.25, 0.630, 0.315, 0.160, and 0.071 mm) plus a bottom pan using a vibratory sieve shaker (Retsch AS-200 Control, Haan, Germany). Sieves and pan were re-weighed with the material retained and expressed as a percentage of initial sample weight. Geometric mean diameter (GMD) and geometric standard deviation (GSD) of the fibrous ingredients were calculated as described by Lyu et al. (2020). Grinding reduced the GMD of OH and SBH by more than 2-fold (Table 2), and virtually eliminated the fraction of coarse particles (>1.25 mm), which is particularly dominant in coarse OH (86%) but also substantial in coarse SH (~40%).

**Table 2.** Particle size distribution (w/w), geometric mean diameter (GMD, μm), and geometric standard deviation (GSD, μm) of coarse and fine soybean hulls (SBH), oat husks (OH), and their mordanted presentation measured using dry sieving^1,2,3,4^

Feed ingredient	Nominal sieve aperture, μm							GMD, μm	GSD, μm
	2,500	1,250	630	315	160	71	<71		
SBH coarse	0.8	39.2	55.6	2.8	0.5	0.4	0.9	1,134	144
SBH coarse mordanted	0.8	2.8	65.5	20.4	6.0	2.7	1.9	692	127
SBH fine	0.0	0.0	20.3	55.8	12.2	5.5	6.2	459	85
SBH fine mordanted	0.1	0.0	9.4	67.1	15.1	6.3	2.2	399	65
OH coarse	27.0	59.1	8.3	2.8	1.3	0.4	1.1	1,686	271
OH coarse mordanted	38.4	45.0	14.4	1.7	0.3	0.1	0.1	1,767	256
OH fine	0.2	0.9	55.1	23.5	7.5	3.2	9.7	649	123
OH fine mordanted	0.3	0.9	48.0	40.6	8.3	1.6	0.4	585	97

^1^Coarse, fibrous ingredients offered unground.

^2^Fine, fibrous ingredients ground in a hammermill with a screen size of 1.0 mm.

^3^GMD, geometric mean diameter; GSD, geometric standard deviation.

^4^GMD (*n*) = 2 replicates.

Water-binding capacity (WBC) of diets and fibers was analyzed in duplicate following the procedure presented by Jacobs et al. (2015), with the following modification: the sample and deionized water were mixed in a conical tube (50 mL) using a vortex mixer at 2,500 rpm for 5 s to guarantee thorough blending of the components.

Bulk density of diets and fibers was analyzed in quintuplicate by fulfilling a 0.5-L graduated cylinder, previously tared, with a representative sample of feeds and fibers. Bulk density (g/L) was calculated as the weight of full cylinder (g) minus the weight of empty cylinder (g) divided by the volume of the cylinder (L).

The physicochemical characterization of the fibrous ingredients and experimental diets is presented in Table 3.

**Table 3.** Water binding capacity (g/g) and bulk density (g/L) of fibrous ingredients and dietary treatments fed to growing pigs^1^^,^^2^^,^^3^^,^^4^

Fibrous ingredients and experimental diets	Physicochemical characteristics	
	Bulk density, g/L	WBC, g/g
SBH coarse	292 ± 5.0	5.5 ± 0.20
SBH coarse mordanted	354 ± 8.5	—
SBH fine	461 ± 9.4	3.8 ± 0.03
SBH fine mordanted	461 ± 8.3	—
OH coarse	195 ± 1.9	2.5 ± 0.20
OH coarse mordanted	217 ± 4.5	—
OH fine	259 ± 7.9	3.5 ± 0.21
OH fine mordanted	226 ± 9.1	—
LF	535 ± 4.7	0.7 ± 0.03
HF	508 ± 3.3	1.2 ± 0.07
HC	483 ± 5.3	1.2 ± 0.06

^1^SBH, soybean hulls; OH, oat husks; LF, Low:Fine; HF, High:Fine; HC, High:Coarse.

^2^WBC, water binding capacity.

^3^Means plus standard deviation for WBC and bulk density.

^4^WBC (*n*) = 2 replicates; bulk density (*n*) = 5 replicates.

### Calculations and statistical analyses

Individual pigs were considered as experimental units. Mean retention time (MRT) of digesta phases and fibers in stomach plus small intestine was calculated using Y_2_O_3_ (solid phase), Co-EDTA (soluble phase), Cr-mordanted (SBH), and Yb-mordanted (OH) as markers. MRT values were estimated for each pig-period-marker combination by non-linear regression procedures (PROC NLIN, SAS 9.4) using a generalized Michaelis–Menten equation (Equation 1) based on van den Borne et al. (2007):


$$\begin{array}{l} y=\left[b0\times c\times t^{\left(-c-1\right)}\times b1^{c}\right]/{\left[1+{\left(\frac{b1}{t}\right)}^{c}\right]}^{2} \end{array}$$ (1)

where *y* is the marker concentration in digesta (g or mg/kg) at time *t* (minutes); b0, b1, and c are parameters that define the curve. Mean retention time was defined as the time of peak and calculated as MRT = [b1^*c*^·(1 − *c*)/( −*c* − 1)]^(1/*c*)^. Digesta phase segregation (min) was calculated as solid digesta MRT − liquid digesta MRT.

Mean retention time data were analyzed using a general linear mixed model (PROC MIXED, SAS 9.4) with MRT as dependent variable, and diet, digesta phase, and their interaction as fixed effects. Digesta fraction (solid, liquid, and fibers) was modelled as within-subjects random (R-side) effect considering an autoregressive covariance AR(1) structure, to account for repeated observations for the various digesta phases within pig. Phase segregation was analyzed using a general linear mixed model (PROC MIXED, SAS 9.4) with phase segregation as dependent variable and diet as fixed effect. Pig was modelled as within-­subjects random (R-side) effect considering an autoregressive ­covariance AR(1) structure, to account for repeated observations within pig.

## Results

Liquid digesta was retained shorter than solid digesta and fiber fractions from the mouth to the terminal ileum, for all treatments (Table 4; *P* < 0.0001). The addition of fibers did not affect MRT of solid (LF: 559 vs HF: 488, *P* = 0.0799) nor liquid digesta (370 vs 411, *P* = 0.2723; Table 4). However, the numerical difference in MRT within solids and liquids among treatments resulted in a significant reduction in digesta phase segregation in HF vs. LF diet (113 min, *P* = 0.0220) with the addition of fibers.

**Table 4.** Mean retention times (MRT, min) and digesta phase segregation (min) estimated from ileal concentrations of indigestible markers observed after a pulse-dose of solid and liquid phase markers, measured in growing pigs fed diets differing in inclusion level and particle size of insoluble fibers^1^^,^^2^^,^^3^^,^^4^

Digesta MRT stomach + small intestine	LF	HF	HC	Pooled SD	*P*-value		
					Digesta phase	Diet	Digesta phase × Diet
Solids (min)	559^A,x^	488^B,x^	497^AB,x^	55.6	<0.001	0.903	0.002
Liquids (min)	370^y^	411^y^	408^y^				
Phase segregation (min)	193^a^	80^b^	85^b^	14.4	—	0.022	—

^1^LSMeans and pooled standard deviation (SD).

^2^Liquids, Co-EDTA; Solids, Y_3_O_2_; Phase segregation (Solids—Liquids).

^3^LF, finely ground low-fiber diet, contained 5 % fine fibers (2.5 % OH + 2.5 % SBH); HF & HC, finely (1mm) ground high-fiber diet and coarse (intact) high-fiber diet, contained 25 % (12.5 % OH + 12.5 % SBH) of fine and coarse fibers, respectively.

^4^LF liquids (*n*) = 5, solids (*n*) = 4, and phase segregation (*n*) = 4; HF liquids, solids, and phase segregation (*n*) = 5; HC liquids, solids, and phase segregation (*n*) = 4.

Superscript letters a, b, A, B indicate differences among columns with *P* < 0.05 or 0.05 < *P* < 0.10, respectively.

Superscript letters x, y indicate differences between solids and liquids within treatment (*P* < 0.05).

Particle size of insoluble fibers did not affect the MRT of solid (488 vs. 497 min, *P* = 0.8128) and liquid digesta (411 vs. 408 min, *P* = 0.9436) between HF and HC, respectively (Table 4); and did not affect digesta phase segregation (80 vs. 85 min, *P* = 0.8437). When detailing the effect of particle size reduction to its main fibrous ingredients using mordanted fibers, grinding of SBH reduced its MRT by almost 100 min (Table 5); while this was not evident for OH (interaction *P* = 0.0146). Furthermore, SBH were retained longer than any other digesta fraction (*P* < 0.05) for both dietary treatments; the OH fraction and the solid phase represented by Y_2_O_3_ did not show differences in MRT between and within treatments (Table 5).

**Table 5.** Mean retention time (MRT, min) of digesta fractions estimated from ileal concentrations of indigestible markers observed over time (0 to 750 min) after a pulse dose of solid, liquid, and mordanted markers, measured in growing pigs fed diets differing in particle size of insoluble fibers^1^^,^^2^^,^^3^^,^^4^

Segment	HF				HC				Pooled SD	P-value		
	Liquids	Solids	Oat husks	Soybean hulls	Liquids	Solids	Oat husks	Soybean hulls		Digesta fraction	Diet	Digesta fraction × Diet
Stomach + Small Intestine	411^d^	488^c^	533^c^	613^b^	408^d^	497^c^	547^bc^	709^a^	50.7	<0.001	0.326	0.015

^1^LSMeans and pooled standard deviation (SD).

^2^HF and HC, finely (1 mm) ground high-fiber diet and coarse (intact) high-fiber diet, contained 25% (12.5% OH + 12.5% SBH) of fine and coarse fibers, respectively.

^3^Liquids = Co-EDTA; solids = Y_3_O_2_; oat husks = Yb mordanted; soybean hulls = Cr mordanted.

^4^High:fine (*n*) = 5/high:coarse (*n*) = 4.

Superscript lettersindicate interaction between marker and treatment (*P* < 0.05).

## Discussion

This study aimed to elucidate the effects of level and particle size of insoluble fibers on digesta transit behavior in the proximal gastrointestinal tract (mouth-terminal ileum) of growing pigs. In a simple monogastric system as in humans and pigs, digesta tends to present a segregated transit through the proximal GIT, where the liquids leave the stomach first, followed by the solids (Holt et al., 1982). Variation in transit of solid and liquid digesta has repercussions on nutrient digestion kinetics (Müller et al., 2018). The rate at which nutrients are digested and absorbed subsequently affects their metabolic use (Boirie et al., 1997). As insoluble fibers may alter digesta transit patterns (Wilfart et al., 2007), a better comprehension about how the addition and the particle size of a blend of insoluble fibers influence the transit of digesta phases can provide valuable insights for enhancing feed efficiency. Thus, this study serves as a model of the effects of insoluble fibers on digesta transit in pigs, acknowledging that the influence of digesta transit on nutrient absorption kinetics may also depend on pigs’ feeding patterns, which can differ among pig types and production systems.

In the present study, the addition of fibers tended to decrease the MRT of solid digesta, and numerically increased the MRT of liquid digesta, together leading to a significant decrease in the segregation of solid and liquid digesta from the mouth to the distal ileum. The addition of 100 g/kg soybean hulls and 100 g/kg oat husks to the diet (corresponding to an increase of 135 g/kg NSP) resulted in a 2.4-fold reduction in digesta phase segregation at the terminal ileum.

In literature, there is no agreement on the effects of insoluble fibers on digesta transit along the proximal GIT. In the study from Ratanpaul et al. (2021), the MRT of solid and liquid digesta increased 72 min (from 288 to 360 min) with the inclusion of 200 g/kg oat husks when compared with 0 and 50 g/kg OH inclusion. In this study, digesta did not present segregation between solids and liquids in terms of MRT. Also, Solà-Oriol et al. (2010) reported that an increase of 166 g/kg NDF by exchanging white rice for oats increased MRT of solid digesta by 102 min. In contrast, Metzler-Zebeli et al. (2019) observed a decrease in digesta MRT of 294 min for solids and 150 min for liquids, when substituting 360 g/kg of maize starch for resistant maize starch. Lastly, Wilfart et al. (2007) found that increasing levels of wheat bran (0, 200, and 400 g/kg) replacing wheat and barley did not affect digesta MRT of solids nor liquids in the stomach but decreased MRT of solids and liquids in the small intestine. The addition of 200 g/kg wheat bran resulted in a reduction in the MRT of liquid digesta from the mouth up to the end of the small intestine by 24 min. This variation in results suggests that the effects of fiber inclusion on digesta behavior depend, among others, on the fibers used and their physicochemical properties (reviewed by Capuano, 2017).

In this study, we analyzed some physicochemical properties of the fibers and diets used as an attempt to better explain the transit behavior observed. Interestingly, the WBC of the high fiber diets was twice as high as that of the low fiber diet regardless of fiber’s particle size. A higher capacity to bind liquids due to higher levels of insoluble fibers could have reduced the transit of liquids, as reported by Takahashi et al. (2009); and consequently led to a decrease in digesta phase segregation. The above is in line with previous results of our group where WBC of diets explained more than 50% of the variation observed in digesta phase segregation in the stomach of growing pigs.

In the current study, although the particle size of both coarse fibrous ingredients was about 2.5 times the particle size of finely ground fibers, particle size did not play a role in modulating MRT up to the distal ileum of solid nor liquid digesta, using Y_2_O_3_ and Co-EDTA as markers, In ­contrast, Wang et al. (2023) observed that fine wheat bran (200 g/kg; 438 μm) increased the MRT of solid digesta (represented by Cr_2_O_3_) in non-pregnant sows by 29 min, compared with coarse wheat bran (605 μm). To our knowledge, this study is the only report available measuring digesta MRT in pigs up to the ileum when adjusting particle size of insoluble fibers. The difference in outcomes between the studies suggests that digesta transit also has been influenced by other factors apart from the particle size contrast we used; among others, the different fibrous ingredients used and physicochemical characteristics of fibers as hardness, morphometry, and rheological properties (Tamargo et al., 2019; Nadia et al., 2021). Several studies have reported that larger and harder food particles are retained longer in the stomach, until their size is reduced to ~2 mm and pass through the pylorus to the small intestine (Bornhorst et al., 2013a, 2013b). Thus, the initial macrostructure of diets and the breakdown rate of its particles play an important role in gastric emptying (Nadia et al., 2021); and gastric maceration leads to a decrease in contrast of particle size in the small intestine (Marciani et al., 2001). However, it is expected that if the particles that reach the small intestine are hard, poorly flexible, and large (like the insoluble fibers), they more easily generate a mat of particles that increases digesta viscosity and stimulates contractile activity and, consequently digesta transit (Lentle and Janssen, 2008). In the current study, this may be an influential factor in the transit behavior difference observed between the SBH and OH. Unfortunately, by using only one cannula at the end of the ileum, it is impossible to observe the behavior of the digesta for the separate segments. Consequently, the obtained results are a sum of what happened in the entire proximal digestive tract.

The addition of mordanted fibers allowed to individually analyze the transit behavior of the 2 main fibrous sources. The use of combined markers has been reported before (Pond et al., 1986; Leterme et al., 1991; Solà-Oriol et al., 2010); however, this technique has not been widely used. The MRT of coarse SBH was greater than that of fine SBH but this was not the case for the OH. The observed variation could be attributed to the morphometry of the fiber sources. Hammermilling of the hard, elongated oat hull particles, typically results in longitudinal size reduction and visual observations confirmed that fine OH particles still had similar length as coarse OH, whereas fine SBH were rather spherical. Unfortunately, the analysis of WBC and bulk density failed to uncover any potential mechanisms associated with the MRT of the individual fibers. Grinding increased the bulk density of SBH to a greater extent compared to OH, yet the MRT of coarse SBH was greater than that of fine SBH. We speculate that other physicochemical parameters of the fibers, as the type of NSP and their interlinkages (Bach Knudsen, 1997), hardness, and disintegration patterns (Kong and Singh, 2009) might be playing a role in the difference in MRT observed between fiber sources.

The disparity between MRT of the OH, SBH, and Y_2_O_3_ allows us to confirm that use of oxides as representative of the transit of solid digesta is not always accurate, particularly when feeding coarse, fibrous ingredients/diets, due to segregation of marker and tracee (de Vries and Gerrits, 2018). The distinct behavior of the mordanted fibers from the finer solids represented by Y_2_O_3_ indicates that the use of different markers, including mordants, provides a better insight of digesta transit behavior than the use of only one type of marker.

## Conclusions

The present study has demonstrated that the addition of soybean hulls (SBH) and oat husks (OH), as insoluble fibers, reduces digesta phase segregation between solids and liquids from the mouth to the distal ileum in growing pigs. This reduction in digesta phase segregation can be related to the increase in water binding capacity observed with higher inclusion levels of these fibers in the diets. Particle size reduction of the insoluble fibers did not affect the MRT of solid nor liquid digesta. These results imply that the addition of insoluble fibers may alter digestion kinetics by altering the transit of digesta phases, regardless of particle size.

It was confirmed that conventional solid markers may not accurately depict the transit patterns of all the solid digesta components, as indicated by the longer MRT of mordanted SBH compared to mordanted OH and solid digesta marker. Coarse SBH were retained longer than fine SBH, whereas OH transit was not affected by the OH particle size.

## Abbreviations:

BW: body weight
CP: crude protein; DM. dry matter
GIT: gastrointestinal tract
GMD: geometric mean diameter
GSD: geometric standard deviation
HF: high-fine diet
HC: high-coarse diet
ICP: inductively coupled plasma
LF: low-fine diet
MRT: mean retention time
N: nitrogen
NE: net energy
NSP: non-starch polysaccharides
OH: oat husks
SBH: soybean hulls
WBC: water binding capacity
