# Correction to: Insoluble fibers affect digesta transit behavior in the upper gastrointestinal tract of growing pigs, regardless of particle size

**DOI:** 10.1093/jas/skad381

**Published:** 2023-11-17

This is a correction to: Sebastian Dorado-Montenegro, Kim Lammers-Jannink, Walter Gerrits, Sonja de Vries, Insoluble fibers affect digesta transit behavior in the upper gastrointestinal tract of growing pigs, regardless of particle size, *Journal of Animal Science*, Volume 101, 2023, skad299, https://doi.org/10.1093/jas/skad299.

In the originally published version of this manuscript, there was an error in the ‘Segment’ column of Table 5, which read ‘Stomach + Stomach’. This has been corrected to ‘Stomach + Small Intestine’.

Additionally, the first and last names of the authors were originally listed in reverse order. This error has also been rectified.

